# Analysis of Risk Factors for Cerebral Microbleeds and the Relationship between Cerebral Microbleeds and Cognitive Impairment

**DOI:** 10.3390/brainsci12111445

**Published:** 2022-10-26

**Authors:** Huiwen Zheng, Yong Yuan, Zuohui Zhang, Jing Zhang

**Affiliations:** 1 Department of Rehabilitation, The Second Affiliated Hospital of Xuzhou Medical University, Xuzhou 221000, China; 2 Department of Neurology, The Affiliated Hospital of Xuzhou Medical University, Xuzhou 221000, China

**Keywords:** cerebral microbleeds, risk factors, cognitive impairment, Amyloid-β1-42, phosphorylated Tau181

## Abstract

(1) Background: Cerebral microbleeds (CMBs) are attracting increasing attention. Nevertheless, the risk factors for CMBs remain poorly identified, and the relationship between CMBs and cognitive impairment is still up for debate; (2) Objective: The present study analyzed the risk factors for CMBs and probed into the potential correlations between the presence, number, and location of CMBs and cognition; (3) Methods: This study enrolled 406 subjects who underwent both brain 3.0-T magnetic resonance imaging scans and cognitive testing. Spearman correlation was used to assess the relationship between the number of CMBs and cognition. Multiple linear regression was utilized to analyze the relationship between the regions of CMBs and each cognitive domain; (4) Results: Multivariate logistic regression analysis results showed that age (odds ratio (*OR*) = 1.045, 95% confidence interval (95%*CI*; 1.009, 1.082)), smoking (*OR* = 3.604, 95%*CI* (1.995, 6.509)), hypertension (*OR* = 3.607, 95%*CI* (2.204, 5.901)), total cholesterol (*OR* = 0.611, 95%*CI* (0.467, 0.799)), and Amyloid-β1-42 (Aβ1-42) (*OR* = 1.028, 95%*CI* (1.018, 1.037)) were the influencing factors of CMBs. Education years (*OR* = 0.959, 95%*CI* (0.930, 0.988)), white matter lesions (*OR* = 2.687, 95%*CI* (1.782, 4.051)), and CMBs (*OR* = 21.246, 95%*CI* (5.728, 21.576)) were the risk factors for cognitive impairment. Hypertension increased the probability of deep CMBs (*OR* = 12.54, 95%*CI* (2.21, 71.28)), while Aβ1-42 elevated the probability of lobar CMBs (*OR* = 1.02, 95%*CI* (1.00, 1.03)). There was a linear correlation between the number of CMBs and Montreal Cognitive Assessment scores (*r* = −0.756, *p* < 0.001). However, CMBs in each region were not related to specific cognitive domains (*p* > 0.05), except CMBs in the mixed group that were negatively correlated with attention (*OR* = −0.669, 95%*CI* (−0.034, −5.270)); (5) Conclusions: Taken together, serum Aβ1-42 levels are related to the presence of CMBs. Cognitive impairment is correlated with the number of CMBs rather than their region. These findings suggest that CMBs play a role in cognitive impairment and that CMBs mark the presence of diffuse vascular injury and neurodegenerative brain damage.

## 1. Introduction

Cognitive impairment has become more burdensome for families and society in recent years due to the increase in life expectancy. Although Alzheimer’s disease is the most common cause of dementia, mounting attention is being paid to cognitive impairment and dementia induced by vascular causes [1]. As imaging technology advances, cerebral small vessel disease (CSVD) is being detected at an increasing rate [2]. As one of the major neuroimaging markers of small vessel disease, cerebral microbleeds (CMBs) have been extensively studied for their risk factors and their association with cognitive impairment [3,4]. Currently, there are no specific drugs to improve cognitive impairment, so early detection and prevention are essential.

Cerebrospinal fluid (CSF) Amyloid-β1-42 (Aβ1-42) and phosphorylated Tau181 (p-Tau181) are well-established hallmarks of Alzheimer’s disease (AD) [5]. Nonetheless, cerebral small vessel disease (CSVD) occasionally coexists with elevations in Aβ and Tau levels, which are the most common causes of cognitive impairments in the elderly [6]. Moreover, prior research has revealed that patients with CSVD can have higher levels of Aβ and p-Tau proteins [6,7]. Additionally, the pathogenesis of CMBs is related to vessel wall damage due to both vascular risk factors and beta-amyloid accumulation [8]. Joseph-Mathurin N et al. stated serum Aβ1-42 as a risk factor for the occurrence of CMBs [9]. As such, it is suggested that CMBs may help explain the overlap of cerebrovascular and neurodegenerative pathologies in cognitive impairment and dementia [3].

Some previous studies have explored the risk factors of CMBs and the correlation of CMBs with cognitive impairment. However, no consensus is yet available on the risk factors of CMBs, especially Aβ1-42 and pTau-181 proteins [6,8]. Likewise, little is known about whether CMBs independently result in cognitive impairment and the effect of their number and region [3,10]. Moreover, further explorations are warranted to dissect the mechanism by which CMBs cause cognitive impairment and their role in vascular injury and neurodegenerative pathology [3,7]. Therefore, our research used susceptibility-weighted imaging (SWI) to accurately detect the number and region of CMBs, thus analyzing the risk factors of CMBs and the relationship between different intracranial conditions and cognitive function in patients with CMBs. In this way, our study aimed to conduct early imaging detection for high-risk patients with CMBs, therefore effectively preventing CMB-related cognitive impairment.

## 2. Materials and Methods

### 2.1. Patient Selection

This study, a case–control study, enrolled 196 patients who were hospitalized in the Department of Neurology of the (second) Affiliated Hospital of Xuzhou Medical University from January 2019 to May 2021 and diagnosed with CMBs through brain magnetic resonance imaging (MRI)-SWI as the CMB group. Additionally, 210 patients without CMBs who were hospitalized in the same period and underwent SWI examination were selected as the non-CMB group. The inclusion criteria: (1) age ranges from 18 to 100 years old; (2) patients who have received brain MRI-SWI after admission and have completed the tests of Mini-Mental State Examination (MMSE) and the Montreal Cognitive Assessment Scale (MoCA). The exclusion criteria of both CMB and non-CMB groups were as follows: (1) patients with acute stroke and brain injury; (2) patients with major brain diseases, such as Alzheimer’s disease, Lewy body dementia, frontotemporal dementia, Parkinson’s disease, tumor, hydrocephalus, trauma, syphilis, AIDS, and Creutzfeldt–Jakob disease; (3) patients who took medications that affect cognitive function and suffered from severe mental diseases; and (4) patients with other imaging changes or diseases that were sufficient to explain cognitive impairment. The study conformed to the Declaration of Helsinki and was approved by the ethics committee of the (second) Affiliated Hospital of Xuzhou Medical University. All patients provided written informed consent prior to enrollment.

### 2.2. Plasma Analyses

All patients fasted for 12 h, and 5 mL of venous blood was collected from the cubital vein of the upper extremity at 8 am the next day for examination. The blood samples were centrifuged at 3000 r/min within 1 h after collection to obtain serum. A double-antibody sandwich enzyme-linked immunosorbent assay (ELISA) was performed with corresponding kits (Shenzhen Anqun Biological Engineering Company, Shenzhen, China) to measure the serum concentrations of Aβ1-42 and p-Tau181. The standard curve was constructed strictly according to the manufacturer’s instructions. Thereafter, the serum concentrations of Aβ1-42 and p-Tau181 were calculated based on the absorbance value of each well.

### 2.3. Brain MRI and SVD Markers

GESIGNAEXCITEHDs3.0-T superconducting whole-body magnetic resonance scanners and 8-channel phased-array head coils were used in this study. All patients were subjected to T1- and T2-weighted imaging (T1WI and T2WI), diffusion-weighted imaging (DWI), fluid-attenuated inversion recovery imaging (FLAIR), and SWI sequences. SWI parameters included a repetition time of 43.6 ms, time of echo of 6 ms, flip angle of 15°, matrix of 350 × 445, field of view of 192 × 220 mm, and slice thickness of 1.2 mm. Imaging was analyzed independently by two professionally trained neuroradiologists, both of whom were blinded to the clinical information of participants. When their conclusions were inconsistent, the classification was conducted after discussion.

**Figure 1:**
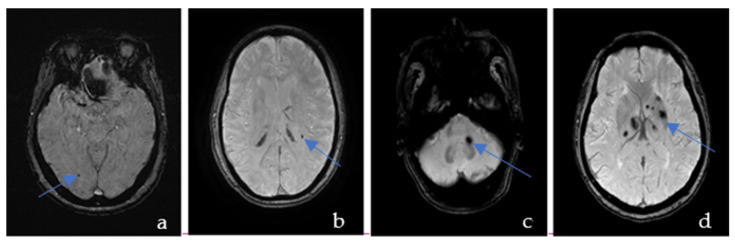
Susceptibility-weighted imaging showing an example of the cerebral microbleeds in the lobar (**a**), deep (**b**), infratentorial (**c**), and mixed regions (**d**).

CMBs are visible as the hypointense ovoid signal on T2-weighted gradient-recalled echo and SWI because of the paramagnetic properties of the blood breakdown product hemosiderin [11]. These lesions may be up to 10 mm in diameter, but the most common diameter is 2–5 mm [12]. According to the Microbleed Anatomical Rating Scale (MARS) [13], CMBs were classified into deep, lobar, and infratentorial CMBs (Figure 1). Lobar regions included cortical and subcortical regions (including subcortical U fibers). Deep regions comprised the basal ganglia, thalamus, internal capsule, external capsule, corpus callosum, and deep and periventricular white matter (DPWM). Infratentorial regions included the brainstem and cerebellum. CMBs distributed in 2 or more areas were named mixed regions. All areas are provided in the anatomical diagram for easy reference. We did not distinguish between definite and possible CMBs but only counted their total numbers as per MARS.

White matter hyperintensities are broadly defined as areas that exhibit high signals in T2-weighted and FLAIR sequences [14]. The Fazekas scale [15,16,17] (0–6 points) was utilized to score the paraventricular and deep white matter lesions (WMLs), respectively, and the scores of the two parts were summed to calculate the total scores. Afterwards, WMLs were ranked in the light of the scores: Grade 0, 0 scores (no WMLs); Grade 1, 1–2 scores (mild WMLs); Grade 2, 3–4 scores (moderate WMLs); and Grade 3, 5–6 scores (severe WMLs).

Lacunes typically appear on FLAIR imaging as 3–15 mm ovoid areas of hypointense signal, usually (though not always) surrounded by a hyperintense rim [11]. The number of lacunes was counted.

Brain atrophy was defined as reduced brain tissue volume, decreased brain parenchyma, flat cerebral gyrus, widened and deepened brain sulci, and enlarged ventricles, cisterns, and subarachnoid spaces. Brain atrophy was assigned into presence and absence groups.

### 2.4. Assessment of Cognitive Function

Cognitive function was assessed with the Beijing version of MMSE and MoCA by physicians blinded to the results of imaging examinations. Both examinations were generally completed once within 15 min and 1 week before and after the SWI examination.

The Beijing version of MMSE [18] included 20 assessment items, which were utilized to examine multiple cognitive domains, such as language, attention, memory, orientation, and calculation. The total score was 30 points, and the cut-off value of the education level was 17 points for the illiterate group, 20 points for the elementary school education group, and 26 points for the secondary school education or higher group.

The MoCA scale [19] comprised seven cognitive domains: (a) visual space and executive function, (b) naming, (c) attention, (d) language, (e) abstract thinking, (f) delayed memory, and (g) orientation. The total score was 30 points. A score greater than 26 was classified as normal cognition. If the years of education were below 12, the evaluation result was increased by 1 point. The higher the score, the better the cognitive function.

### 2.5. Vascular Risk Factors

Hypertension was defined as a systolic blood pressure of ≥140 mmHg and/or a diastolic blood pressure of ≥90 mmHg or the use of blood pressure-lowering medication. Individuals were considered diabetic when their fasting blood glucose level was ≥7.0 mmol/L or when they used glucose-lowering medication. Smoking and drinking behaviors were categorized as “ever” or “never” smoking or drinking. A “Drinker” refers to females consuming alcohol over 50 g per day and males drinking alcohol over 60 g per day. Medication use (glucose-lowering, blood pressure-lowering, and lipid-lowering medication) and education levels were recorded during medical history collection [6].

### 2.6. Statistical Analysis

SPSS 22.0 software was utilized for statistical analysis of relevant data. Normal distribution was tested, and the quantitative data conforming to normal distribution were summarized as mean ± standard deviation. The independent-sample *t*-test was used to compare data between two groups. The data that did not obey a normal distribution were expressed as the median (quartile), and the Mann–Whitney *U* test was utilized for comparisons between two groups. Qualitative data were all presented as counts and percentages, and comparisons between two groups were analyzed with the χ^2^ test. The factors with *p* < 0.05 tested by χ^2^ were included in the multivariate regression analysis. A binary logistic regression analysis was used to identify the influencing factors of CMBs and cognitive impairment. A disordered multi-class logistic regression analysis was conducted to analyze the risk factors of CMBs in different regions (lobar, deep, infratentorial, and mixed regions). In the cognitive impairment group, Spearman correlation was utilized to determine the correlation between the number of CMBs and MoCA scores. Multiple linear regression was used to analyze the relationship of the regions of CMBs with MoCA scores and each cognitive domain of MoCA by setting dummy variables.

## 3. Results

### 3.1. Analysis of Risk Factors of CMBs

#### 3.1.1. The Univariate Analysis of the Risk Factors for CMBs

Of the 406 participants, 196 patients suffered from CMBs. There was no statistically significant difference between the CMBs group and the non-CMBs group in terms of gender, years of education, the number of drinkers, history of diabetes, coronary heart disease, the proportion of individuals using lipid-lowering medications, and the levels of triglyceride (TG), high-density lipoprotein cholesterol (HDL-C), low-density lipoprotein cholesterol (LDL-C), and blood uric acid (BUA) (*p* > 0.05). In addition, subjects in the CMBs group were older than those in the non-CMBs group. Meanwhile, the smoking rate, the proportion of individuals with a history of hypertension and the use of antithrombotic drugs, and the levels of Hcy, Aβ1-42, and pTau-181 were higher in the CMBs group than in the non-CMBs group, accompanied by lower total cholesterol (TC) levels (*p* < 0.05; Table 1).

#### 3.1.2. The Multivariate Analysis of the Risk Factors for CMBs

The variables with *p* < 0.05 tested by χ^2^ were utilized as the independent variable and the presence and absence of CMBs as the dependent variable (assignment: yes = 1, no = 0) to conduct the multivariate logistic regression analysis (the forward Wald method). The results manifested that age, smoking, history of hypertension, TC, and Aβ1-42 were the influencing factors of CMBs (*p* < 0.05; Table 2).

#### 3.1.3. The Risk Factors for CMBs in Different Regions

A multinomial logistic regression model was constructed with the common risk factors as independent variables and CMBs in different regions as dependent variables. The results are listed in Table 3.

#### 3.1.4. The Multivariate Analysis of the Risk Factors for CMBs in Different Regions

A multinomial logistic regression model was constructed with the meaningful indicators in Table 3 as independent variables and CMBs in different regions as dependent variables. The results are displayed in Table 4.

### 3.2. Analysis of the Relationship between CMBs and Cognitive Impairment

#### 3.2.1. The Univariate Analysis of the Characteristics of Cognitive Impairment

No statistically significant difference was observed in gender, smoking rate, alcohol consumption rate, the proportion of individuals with a history of diabetes, coronary heart disease, and use of antithrombotic drugs and lipid-lowering drugs, and the levels of TG, HDL-C, LDL-C, and BUA between the cognitive impairment group and the non-cognitive impairment group (*p* > 0.05). Subjects were older in the cognitive impairment group than in the non-cognitive impairment group. In addition, the cognitive impairment group had shorter years of education, a higher proportion of individuals with a history of hypertension, higher Hcy, Aβ1-42, and pTau-181 levels, and lower TC levels than the non-cognitive impairment group (*p* < 0.05; Table 5).

#### 3.2.2. The Univariate Analysis of the Imaging Characteristics of Cognitive Impairment

Imaging characteristics of the study population grouped in accordance with the presence and absence of cognitive impairment. There were statistical differences in the distribution of CMBs and WMLs between the cognitive impairment group and the non-cognitive impairment group (Table 6).

#### 3.2.3. The Multivariate Analysis of the Risk Factors for Cognitive Impairment

Multivariate logistic regression analysis (the forward LR method) was conducted with the factors, with *p* < 0.05 tested by χ^2^ as independent variables and the presence and absence of cognitive impairment as the dependent variable. The results demonstrated years of education, WML, and CMBs as the risk factors for patients with cognitive impairment (Table 7).

#### 3.2.4. The Relationship between the Number of CMBs and MoCA Scores

**Figure 2:**
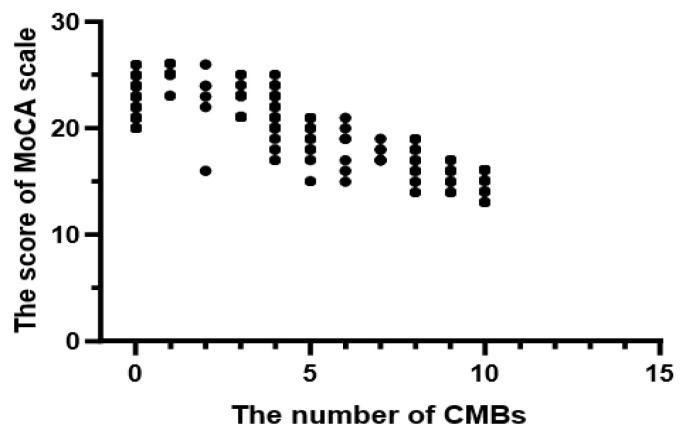
The scatter diagram of the number of CMBs and MoCA scores.

In the cognitive impairment group, the number of CMBs was 3 (0, 6) and the MoCA score was 20.7 ± 3.42 points. The scatter diagram is depicted in Figure 2. Spearman correlation results exhibited a linear correlation between the number of CMBs and MoCA scores (r = −0.756, *p* < 0.001).

#### 3.2.5. The Relationship between CMBs in Different Regions and MoCA Scores

In the cognitive impairment group, the input method was utilized to construct the multiple linear regression model1, with the MoCA score as the dependent variable, CMBs in different regions as the independent variable, and patients without CMBs as reference variables. The results manifested that lobar, deep, infratentorial, and mixed CMBs were all risk factors influencing MoCA scores. After correction for the effects of age, years of education, WML, and the number of CMBs, CMBs in the lobar and mixed regions remained the risk factors influencing MoCA scores (Table 8).

#### 3.2.6. The Relationship between CMBs in Different Regions and Specific Cognitive Domains

**Figure 3:**
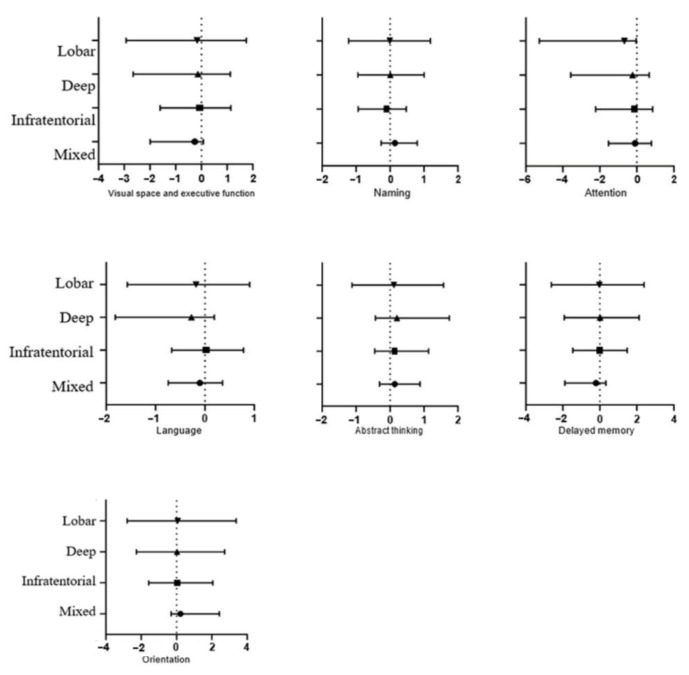
The relationship between CMBs in different regions and specific cognitive domains. Note: The x-axis represents the specific cognitive domains for the categories of lobar, deep, infratentorial, and mixed CMBs (y-axis), compared with a reference group without cerebral microbleeds. Error bars represent 95% confidence intervals.

In the cognitive impairment group, a multiple linear regression model was generated by the input method, with the individual cognitive domain scores of MoCA as dependent variables, age, years of education, WML, the number of CMBs, and CMBs in different regions as independent variables, and patients without CMBs as reference variables. These results might not be exactly right due to the small sample size. After correction for the influences of age, years of education, WML, and the number of CMBs, the results showed that CMBs in each region might not be related to specific cognitive domains except for CMBs in the mixed group, which were negatively correlated with attention (Figure 3).

## 4. Discussion

Increasing cases of CMBs have been detected with the advancement of imaging diagnosis technology. In the general population, the prevalence of CMBs is approximately 15.3% [3] and gradually increases as people age [20]. The risk factors of CMBs and their relationship with cognitive impairment have been extensively studied. However, there is currently no consensus on the risk factors of CMBs, especially Aβ1-42 and pTau-181 proteins. Our study is the first study in China comprehensively evaluating the questions mentioned above. Our current study included a total of 406 patients with CMBs and investigated the risk factors for CMBs and the association between CMBs and cognitive impairment.

Cerebral amyloid angiopathy (CAA) predominantly affects cortical arteries and hence is characterized by lobar microbleeds, whilst hypertensive arteriopathy typically influences small perforating end arteries in deep brain areas and is featured by deep microbleeds [7]. In a cross-sectional study of the elderly in Framingham, low total cholesterol levels increased the risk of lobar CMBs, statin use increased the risk of lobar and mixed CMBs, and the association was not affected by adjustment for cholesterol levels or concomitant medication use. Nevertheless, a prior meta-analysis did not demonstrate such an association [21]. Another meta-analysis of 20,988 participants from 37 studies showed that CMBs were more frequent in antiplatelet users than in non-antiplatelet users and that antiplatelet therapy was significantly associated with lobar CMBs rather than deep or infratentorial CMBs [22]. Although CMBs are a typical feature of CAA [23] and Aβ deposits and p-Tau proteins are typical pathological markers of degenerative diseases, especially Alzheimer’s disease [24], accumulating studies have elucidated that Aβ proteins, p-Tau proteins, and CSVD, which occasionally coexist, are the most common reason for cognitive impairment in the elderly [6].

Prior studies have unraveled that CSVD can contribute to the upregulation of Aβ and p-Tau proteins [6,7]. Moreover, any combination of concurrent lobar and deep microbleeds illustrates hypertensive angiopathy [25]. This provides conditions for us to investigate the relationship of CMBs with Aβ and p-Tau proteins. Our study found that subjects with CMBs were older than subjects without CMBs and that the smoking rate, the proportion of individuals with a history of hypertension and use of antithrombotic drugs, and the levels of Hcy, Aβ1-42, and p-Tau181 were higher and TC levels were lower in the CMB group than in the non-CMB group, consistent with previous studies. However, no statistically significant difference was found in the use of antiplatelet drugs and the levels of Hcy and p-Tau181 after multivariate logistic regression analyses. We suspect that the occurrence and progression of CMBs are not directly triggered by these factors and may be correlated with some known or unknown indirect effects. As discussed in one article, CMBs may represent not only the damage of a certain blood vessel but also a downstream product of both severe vascular and neurodegenerative pathologies [3]. In the univariate and multivariate analyses of CMBs in different regions, it was found that aging elevated the risk of CMBs in each region. Additionally, we also observed that hypertension mainly enhanced the risk of deep CMBs, and that Aβ1-42 protein upregulation increased the risk of lobar CMBs. It is still unclear whether elevated Aβ1-42 protein is related to CAA, but research on CAA has elaborated that CMBs resulting from CAA are majorly associated with Aβ_1-40_. Strictly, lobar CMBs only accounted for 16.5% of our research subjects. Consequently, there is no longer a clear distinction between vascular pathology and degenerative diseases.

Accumulating studies have reported that CMBs are related to a decline in cognitive function [3,26,27,28]. A longitudinal study on the general population unveiled that participants with more than three CMBs, regardless of their locations, had a higher incidence of all-cause dementia and vascular dementia [29]. A study conducted by Chung et al. on 959 elderly people in the community revealed that strictly lobar, but not deep or infratentorial, CMBs were associated with changes in cognitive function, especially visuospatial executive function [10]. A study of Wang et al. on patients with cerebral infarction/transient ischemic attack demonstrated that attention deficits are particularly prominent in patients with deep CMBs [30]. Nevertheless, a prior study has also manifested that mixed CMBs or a higher load of CMBs with some specificity for location is correlated with accelerated cognitive function decline in the elderly [29]. In short, there remains poor identification of the relationship between the number and location of CMBs with overall cognitive function and various cognitive domains. In this study, age was older in the cognitive impairment group than in the non-cognitive impairment group, accompanied by shorter years of education, a higher proportion of people with a history of hypertension, higher Hcy, Aβ1-42, and pTau-181 levels, and lower TC levels. Conversely, after multivariate analyses, only years of education, CMBs, and WMLs were the independent risk factors for cognitive impairment, concurrent with previous research [26]. These results suggested an independent role of microbleed-associated vasculopathy in cognitive impairment and that CMBs were an independent risk factor for cognitive impairment. Then, a correlation analysis was conducted to clarify the relationship between the number of CMBs and MoCA scores, and the data indeed demonstrated that the overall cognitive function of patients worsened as the number increased. Multiple linear regression analysis results uncovered that CMBs in each region were correlated with a decline in overall cognitive function. After the influence of other meaningful variables was adjusted, only lobar and mixed CMBs were statistically significantly associated with a decline in cognitive function. Likewise, each cognitive domain was also analyzed, which showed that CMBs in specific regions were unrelated to the damage of specific cognitive domains, concordant with previous observations [29]. It has been previously reported that both WML and lacuna elevated the prevalence of CMBs [30,31,32]. Combined with our research results, we speculate that cognitive impairment caused by CMBs is not mainly attributed to the destruction of local cortical function and brain network structure but marks the presence of diffuse vascular injury and neurodegenerative brain damage.

Although CMBs play an independent role in the risk of cognitive impairment, the mechanism is still controversial. The question is how vascular pathology interacts with amyloid pathology to cause clinical cognitive deterioration. Vascular diseases can result in reductions in amyloid clearance and deposition, and hemorrhagic and ischemic changes can occur when amyloid acts on blood vessels [3]. Some previous studies have demonstrated that CMBs in patients with cognitive function decline may present several features of blood-brain barrier dysfunction [33,34,35]. A study on Tau proteins suggested that both Aβ and CSVD were independently associated with increased Tau accumulation and that Tau burden plays a pivotal role since it was the final common pathway for cognitive impairment in patients with subcortical vascular cognitive impairment [6]. Conclusively, CMBs are closely related to Aβ1-42 and pTau-181, and they often coexist and cooperate in impairing cognitive function.

Several limitations deserve consideration. First, our research is a cross-sectional study without follow-up observation. In this context, we were not able to obtain the longitudinal data of the patients. Therefore, the cross-sectional design of this analysis limits the inference of the causal relationship between CMBs and cognitive impairment. Secondly, due to the limitation of conditions, we only obtained the data on serum Aβ_1-42_ protein levels, which precluded us from comparing the relationship between Aβ_1-42_, Aβ_1-40_, and Aβ_1-38_ in different regions of CMBs. Thirdly, selection bias between the two groups might be introduced when the two groups were established. Fourth, some important potential confounders, such as atrial fibrillation, have not been considered in this analysis. Finally, our research population is limited to patients with CSVD, and our results and values are no longer applicable to patients with other diseases and the general population.

## Figures and Tables

**Table 1:** Characteristics of the study population grouped according to the presence or absence of CMBs.

Clinical Characteristics	Non-CMBs (*n* = 210)	CMBs (*n* = 196)	*t*/χ^2^ Value	*p*-Value
Men/Women	151/59	140/56	0.011	0.915
Age (mean ± SD), years	68.10 ± 6.54	69.93 ± 6.87	−2.751	0.006
Education (mean ± SD), years	7.01 ± 2.62	6.89 ± 3.44	1.801	0.075
Smoker (*n* (%))	29(13.80)	55(28.06)	12.549	<0.001
Drinker (*n* (%))	28(13.33%)	25(12.76%)	0.030	0.863
Hypertension (*n* (%))	87(41.43%)	148(75.51%)	23.433	<0.001
Diabetes (*n* (%))	42(20.00%)	37(18.88%)	0.082	0.775
CHD (*n* (%))	33(15.71%)	45(22.96%)	3.428	0.064
Antithrombotic medication	52(24.76%)	74(37.76%)	7.997	0.005
Lipid-lowering medication	36(17.14%)	22(11.22%)	0.816	0.366
TC (mean ± SD)	4.08 ± 1.02	3.66 ± 0.81	4.618	<0.001
TG (mean ± SD)	1.48 ± 0.49	1.68 ± 1.38	−1.811	0.061
HDL-C (mean ± SD)	1.13 ± 0.31	1.08 ± 0.29	1.819	0.070
LDL-C (mean ± SD)	2.39 ± 0.94	2.37 ± 0.77	0.203	0.404
BUA (mean ± SD), μmol/L	301.30 ± 79.56	312.37 ± 79.73	−1.399	0.400
HCY (mean ± SD), μmol/L	16.93 ± 8.35	20.20 ± 10.17	−3.552	<0.001
Aβ1-42(mean ± SD), μmol/L	44.75 ± 24.80	70.71 ± 37.49	−8.171	<0.001
pTau-81(mean ± SD), μmol/L	21.29 ± 12.67	25.17 ± 10.10	−3.396	0.001

**Table 2:** The results of the multivariate analysis of the risk factors for CMBs.

Variable	*B*	*SE*	*Ward*	*p*	*OR* (95%*CI*)
Age	0.044	0.018	6.030	0.014	1.045 (1.009, 1.082)
Hypertension	1.283	0.251	26.070	<0.001	3.607 (2.204, 5.901)
Smoking	1.282	0.302	18.059	<0.001	3.604 (1.995, 6.509)
Antithrombotic medication	0.068	0.272	0.062	0.803	1.070 (0.628, 1.822)
TC	−0.493	0.137	12.966	<0.001	0.611 (0.467, 0.799)
HCY	0.010	0.014	0.498	0.481	1.010 (0.982, 1.039)
Aβ1-42	0.027	0.005	34.876	<0.001	1.028 (1.018, 1.037)
pTau-181	−0.010	0.011	0.788	0.375	0.990 (0.969, 1.012)

Note: TC (Total cholesterol), HCY (Homocysteine).

**Table 3:** The analysis of the risk factors for CMBs in different regions.

	Lobar CMBs (*n* = 67) *OR* (95%*CI*)	Deep CMBs (*n* = 47) *OR* (95%*CI*)	Infratentorial CMBs (*n* = 27) *OR* (95%*CI*)	Mixed CMBs (*n* = 55) *OR* (95%*CI*)
Men	1.10 (0.44–2.73)	0.93 (0.33–2.64)	0.93 (0.23–3.84)	0.97 (0.37–2.57)
Age	1.12 (1.00–2.12)	1.08 (1.01–1.16)	1.32 (1.10–1.44)	1.11 (1.00–1.20)
Smoking	2.55 (0.95–6.81)	4.67 (1.62–13.41)	1.75 (0.34–9.09)	2.33 (0.79–6.85)
Drinking	1.50 (0.53–4.24)	1.06 (0.28–4.03)	2.57 (0.60–10.99)	1.34 (0.60–3.29)
Hypertension	0.89 (0.39–2.02)	12.00 (2.66–54.21)	5.33 (1.08–26.26)	2.67 (1.06–6.74)
Diabetes	1.02 (0.37–2.81)	0.72 (0.19–2.68)	0.46 (0.06–3.78)	1.36 (0.48–3.84)
CHD	2.24 (0.88–5.67)	1.74 (0.56–5.39)	2.24 (0.53–9.48)	1.37 (0.45–4.16)
Antithrombotic medication	0.91 (0.38–2.17)	0.37 (0.10–1.35)	0.24 (0.03–1.92)	1.27 (0.51–3.17)
Lipid-lowering medication	2.44 (1.04–5.77)	0.65 (0.18–2.39)	0.92 (0.18–4.60)	1.51 (0.56–4.06)
TC	0.43 (0.24–0.75)	0.49 (0.26–0.92)	0.61 (0.27–1.35)	0.82 (0.51–1.32)
TG	1.78 (1.07–2.97)	1.98 (1.18–3.34)	1.09 (0.37–3.23)	1.79 (1.06–3.04)
HDL-C	0.29 (0.07–1.24)	0.16 (0.03–0.99)	0.21 (0.02–2.34)	0.34 (0.07–1.61)
LDL-C	0.91 (0.56–1.47)	0.89 (0.50–1.58)	1.04 (0.49–2.19)	1.14 (0.69–1.87)
BUA	1.00 (0.99–1.01)	1.00 (0.99–1.01)	1.00 (0.99–1.01)	1.00 (0.99–1.01)
HCY	1.07 (1.03–1.12)	1.05 (0.99–1.09)	1.05 (0.98–1.11)	0.98 (0.91–1.05)
Aβ_1-42_	1.02 (1.01–1.03)	1.00 (0.98–1.02)	1.02 (1.00–1.03)	1.01 (1.00–1.03)
pTau-181	1.04 (1.01–1.07)	1.00 (0.95–1.05)	1.06 (1.02–1.11)	1.03 (0.99–1.07)

Note: CHD (coronary heart disease), TC (Total cholesterol, TG (Triglyceride), HDL-C (High-density lipoprotein cholesterol), LDL-C (Low-density lipoprotein cholesterol), BUA (Blood uric acid), HCY (Homocysteine).

**Table 4:** Multivariate analysis results of the risk factors for CMBs in different regions.

	Lobar CMBs (*n* = 67) *OR* (95%*CI*)	Deep CMBs (*n* = 47) *OR* (95%*CI*)	Infratentorial CMBs (*n* = 27) *OR* (95%*CI*)	Mixed CMBs (*n* = 55) *OR* (95%*CI*)
Age	1.02 (0.94–1.10)	1.13 (1.04–1.24)	0.95 (0.83–1.08)	1.04 (0.97–1.12)
Smoking	2.35 (0.62–8.89)	6.02 (1.53–23.71)	0.40 (0.03–5.65)	2.37 (0.70–7.95)
Hypertension	0.64 (0.24–1.71)	12.54 (2.21–71.28)	4.40 (0.78–24.81)	2.11 (0.78–5.70)
Antithrombotic	0.01 (0.00–0.29)	0.15 (0.02–1.40)	1.48 (1.04–2.39)	0.43 (0.07–5.70)
medication				
Lipid-lowering	9.46 (4.20–20.65)	2.55 (0.22–28.94)	3.48 (1.44–8.39)	3.27 (0.52–20.79)
medication				
TC	0.26 (0.13–0.54)	0.26 (0.12–0.55)	0.52 (0.19–1.40)	0.59 (0.32–1.06)
TG	2.57 (1.14–5.83)	4.48 (1.94–10.39)	1.19 (0.31–4.59)	2.91 (1.29–6.59)
HDL-C	0.64 (0.10–4.17)	1.28 (0.12–13.64)	0.19 (0.01–5.86)	0.99 (0.15–6.69)
Aβ1-42	1.02 (1.00–1.03)	1.01 (0.99–1.03)	1.02 (0.99–1.04)	1.01 (0.99–1.03)
pTau-181	0.99 (0.95–1.04)	0.95 (0.89–1.02)	1.07 (0.99–1.15)	0.99 (0.95–1.05)

Note: TC (Total cholesterol), TG (Triglyceride), HDL-C (High-density lipoprotein cholesterol).

**Table 5:** Characteristics of the study population grouped according to the presence and absence of cognitive impairment.

	Cognitive Impairment (*n* = 160)	Non-Cognitive Impairment (*n* = 246)	*t*/χ^2^	*p*-Value
Men/Women	115 (71.9%)	176 (71.5%)	0.005	0.942
Age, years (mean ± SD)	70.07 ± 6.71	68.45 ± 6.45	2.429	0.016
Education, years (mean ± SD)	5.84 ± 2.81	6.75 ± 2.96	−3.085	0.002
Smoker (*n* (%))	32 (20.0%)	52 (21.1%)	0.077	0.782
Drinker (*n* (%))	18 (20.0%)	35 (14.2%)	0.518	0.384
Hypertension (*n* (%))	103 (64.4%)	132 (53.7%)	4.567	0.033
Diabetes (*n* (%))	34 (21.3%)	44 (17.9%)	0.709	0.400
CHD (*n* (%))	33 (20.6%)	45 (18.3%)	0.707	0.401
Antithrombotic medication (*n* (%))	48 (30.0%)	78 (31.7%)	0.132	0.716
Lipid-lowering medication (*n* (%))	22 (13.8%)	36 (14.6%)	0.062	0.804
TC, mmol/L (mean ± SD)	3.74 ± 0.91	3.96 ± 0.96	−2.340	0.020
TG, mmol/L (mean ± SD)	1.67 ± 1.30	1.52 ± 0.79	1.350	0.178
HDL-C, mmol/L (mean ± SD)	1.01 ± 0.31	1.12 ± 0.30	−0.704	0.482
LDL-C, mmol/L (mean ± SD)	2.35 ± 0.82	2.40 ± 0.89	−0.554	0.580
BUA μmol/L (mean ± SD)	310.01 ± 81.50	304.50 ± 78.65	0.685	0.494
HCY, μmol/L (mean ± SD)	18.86 ± 8.92	17.04 ± 7.91	2.093	0.037
Aβ1-42, pg/mL (mean ± SD)	65.25 ± 36.87	52.10 ± 31.17	3.726	<0.001
pTau-81, pg/mL (mean ± SD)	26.07 ± 10.99	22.24 ± 11.99	3.244	0.001

Note: CHD (coronary heart disease), TC (Total cholesterol), TG (Triglyceride), HDL-C (High-density lipoprotein cholesterol), LDL-C (Low-density lipoprotein cholesterol), BUA (Blood uric acid), HCY (Homocysteine).

**Table 6:** Imaging characteristics of the study population grouped according to whether subjects experienced cognitive impairment.

	Cognitive Impairment (*n* = 160)	Non-Cognitive Impairment (*n* = 246)	χ^2^/Z	*p*-Value
**Lacunes**			4.308	0.516
0	16	30	*OR*	95%*CI*
1–5	90	116	1.455	0.747–2.832
6–10	27	59	0.858	0.402–1.832
>10	27	41	1.235	0.568–2.686
**CMBs**			58.623	<0.001
None	48	162	*OR*	95%*CI*
Lobar	37	30	4.162	2.332–7.429
Deep	22	25	2.970	1.539–5.731
Infratentorial	13	14	3.134	1.379–7.121
Mixed	40	15	9.001	4.582–17.680
**WML**			9.829	<0.001
0	69	165	*OR*	95%*CI*
1	43	46	2.235	1.353–3.692
2	25	20	2.989	1.558–5.735
3	23	15	3.667	1.805–7.447
**Brain atrophy**			0.869	0.351
inexistence	124	200	*OR*	95%*CI*
existence	36	46	1.262	0.773–2.061

Note: CMBs (Cerebral microbleeds), WML (White Matter Lesions).

**Table 7:** Multivariate analysis results of the risk factors for cognitive impairment.

	*B*	*SE*	*WALD*	*p*	*OR* (95%*CI*)
Age	0.032	0.019	2.729	0.099	1.032 (0.994, 1.071)
Education years	−0.040	0.014	7.910	0.005	0.959(0.930, 0.988)
Hypertension	0.212	0.257	0.681	0.409	1.237(0.747, 2.048)
TC	−0.168	0.130	1.669	0.196	0.846 (0.656, 1.091)
HCY	0.086	0.050	3.000	0.083	0.918 (0.833, 1.011)
AΒ1-42	0.004	0.004	0.778	0.378	1.004 (0.996, 1.012)
pTau-181	−0.015	0.012	1.686	0.194	0.985 (0.962, 1.008)
WML	0.988	0.209	22.25	<0.001	2.687 (1.782, 4.051)
CMBs					
Lobar	3.071	0.677	20.608	<0.001	21.246 (5.728, 21.576)
Deep	3.288	0.812	16.418	<0.001	26.798 (5.462, 131.488)
Infratentorial	3.297	0.842	15.342	<0.001	27.028 (5.193, 140.690)
Mixed	4.811	0.806	35.632	<0.001	122.884 (25.317, 596.446)

Note: TC (Total cholesterol), HCY (Homocysteine), CMBs (Cerebral microbleeds), WML (White Matter Lesions).

**Table 8:** The relationship between CMBs in different regions and MoCA scores.

**Model 1**				
**Regions**	**Standardized**	***β***	***p*** **-Value**	**95%*CI***
Lobar	−0.487		<0.001	(−5.278, −2.605)
Deep	−0.231		0.005	(−3.861, −0.715)
Infratentorial	−0.301		<0.001	(−5.666, −1.846)
Mixed	−0.306		<0.001	(−3.716, −1.100)
**Model 2**				
**Regions**	**Standardized**	***β***	***p*** **-Value**	**95%*CI***
Lobar	−0.375		<0.001	(−4.188, −1.884)
Deep	−0.201		0.081	(−0.772, 5.613)
Infratentorial	−0.216		0.072	(−0.211, 4.814)
Mixed	−0.292		<0.001	(−4.814, −0.211)

Note: Model 2 was obtained after correcting for the effects of WML, CMB number, age, and education years. CMBs (Cerebral microbleeds), WML (White Matter Lesions).

## Data Availability

Not applicable.
